# Minor surgical procedures during immune tolerance induction in people with hemophilia A and inhibitors: results from the Brazilian Immune Tolerance (BrazIT) study cohort

**DOI:** 10.1016/j.htct.2024.05.010

**Published:** 2024-09-10

**Authors:** Ricardo Mesquita Camelo, Maíse Moreira Dias, Suely Meireles Rezende, Brazilian Immune Tolerance (BrazIT) Study group

**Affiliations:** a Faculty of Medicine, Universidade Federal de Minas Gerais, Belo Horizonte, Brazil; b Department of Clinical Epidemiology, Leiden University Medical Centre, Leiden, the Netherlands

**Keywords:** Hemophilia A, Inhibitors, Immune tolerance induction, Surgery, Outcome

## Abstract

**Introduction:**

Surgeries are implicated in the development of anti-factor VIII (FVIII) neutralizing antibodies (inhibitors) in hemophilia A individuals with immune tolerance induction (ITI) treatment being the recommended therapy to eradicate these inhibitors. We evaluated the association of surgical procedures performed during ITI and treatment outcome.

**Methods:**

Patients were treated according to the Brazilian ITI Protocol with outcomes being defined as successful (i.e., recovered responsiveness to exogenous FVIII) and failed (i.e., unresponsiveness to exogenous FVIII thus requiring bypassing agents for bleeding control). Surgical procedures during induction therapy were managed following international recommendations.

**Results:**

Treatment success rate was 68.7 % in 163 patients; 33 (20.2 %) were submitted to 43 (96 %) minor and two major surgeries. Personal, hemophilia, inhibitor, and treatment characteristics were similar between patients submitted to surgical procedures or not while on ITI; the success rates were 72.7 % and 67.7 % (p-value = 0.577), respectively.

**Conclusion:**

No association was found between having a minor surgical procedure and ITI treatment outcome.

## Introduction

Hemophilia A is a rare bleeding disorder due to reduced or even absent clotting factor VIII (FVIII) activity. People with hemophilia A require FVIII replacement to both control (episodic treatment) and avoid (prophylaxis) bleeding.^1^ However, the development of neutralizing anti-FVIII antibodies (inhibitors) renders individuals unresponsive to exogenous FVIII.^1^ In this case, bypassing agents are required for episodic or prophylactic management of bleeding or emicizumab as prophylaxis.^1^ It is known that intensive treatment with FVIII (high doses and long exposures), including during surgical procedures, is a risk factor for inhibitor development.^2^^,^^3^ Inflammatory imbalance during surgeries may trigger an immune response against exogenous FVIII.^3^^,^^4^

Immune tolerance induction (ITI), consisting of frequent infusions of exogenous FVIII to induce (re)tolerance, is the treatment of choice to eradicate inhibitors.^1^ Response to induction varies with success rates ranging from 60 to 90 %.^1^^,^^5^ The main determinants of the outcome are inhibitor-related, such as the inhibitor titer peaks before and during induction.^6^ The Brazilian ITI (BrazIT) Study, a multicenter cohort of patients who completed ITI, was set up to further explore predictors of outcome.^5^ The current paper reports on a subset of the BrazIT Study. We hypothesized that the immunological imbalance that causes inhibitor development during surgical procedures^2^, ^3^, ^4^ might also influence the ITI outcome. Therefore, the ITI outcomes of all participants of the BrazIT Study who underwent surgical procedures were evaluated.

## Methods

This study was approved centrally (CAAE 52812415.8.0000.5149) and locally at 15 Brazilian hemophilia treatment centers. Healthcare professionals involved in the study enrolled patients from June 2016 to January 2021 before initiating, during, or after completing ITI, after obtaining signed consent from the patients or their guardians. ITI treatment started from April 2010 to August 2019. Patients (*n* = 166) who had completed ITI by January 2022 were included in the current analysis. Subsequently, three patients who had no information about their surgical procedures were excluded.

All patients were treated according to the Brazilian ITI Protocol. Briefly, patients of any age, disease severity, or inhibitor response were offered ITI if they had an active inhibitor which resulted in the patient being unresponsive to FVIII. The FVIII concentrate used for induction was the type (either plasma-derived or recombinant) against which the patient had developed antibodies. Treatment started with low doses (50 IU/kg 3x weekly) however, a higher dose (100 IU/kg daily) was prescribed when unresponsive after six months of therapy. Prophylaxis with bypassing agents was prescribed at the physician's discretion. Follow-up included monthly inhibitor analyses at each center according to the protocol. Response to ITI was based on inhibitor titer, FVIII pharmacokinetics, and responsiveness to FVIII. Inhibitor titers <0.6 Bethesda units/mL (BU/mL) were considered negative. ITI outcomes were defined as successful (i.e., recovered responsiveness to exogenous FVIII) or failed (i.e., no responsiveness to exogenous FVIII thus requiring bypassing agents for bleeding control). The recommended maximum ITI duration was 33 months however, in some cases therapy lasted longer due to individual characteristics. The genotypes of the FVIII-encoding gene (*F8*) were grouped as high-risk (large deletions, inversions, and nonsense mutations) or low-risk (small insertions/deletions and missense mutations) for inhibitor development.^7^

Surgical procedures were indicated and managed by an interdisciplinary team that comprised at least one surgeon and one hematologist who was a specialist in hemophilia.^1^ Both elective and urgent surgeries followed international recommendations for clotting factor coverage.^1^ Surgeries were classified as minor or major as detailed in a previous report.^8^ ITI was not ceased during the procedures.

## Results

Most (*n* = 145; 91.2 %) individuals had severe hemophilia A (Table 1) with high-risk mutations being the most prevalent (90.9 %) and 157 (96.3 %) had high-responding inhibitors. The median age at the initiation of induction was 7.0 years (interquartile range [IQR]: 2.5–19.7 years) with a median duration of 2.6 years (IQR: 1.7–3.1 years). ITI was longer for those who failed (3.1 years; IQR: 2.6–3.6 years) compared to those who had successful induction (2.2 years; IQR: 1.5–2.9 years; p-value < 0.001) with the overall success rate being 68.7 %.

**Table 1:** Characteristics of people with hemophilia A on immune tolerance induction who underwent surgery or not.

Characteristic	Total (*n* = 163)	Missing data	Surgery (*n* = 33)	No surgery (*n* = 130)	*p*-value
Patient and hemophilia characteristics					
Male n (%)	162 (99.4 %)	0	33 (100.0 %)	129 (99.2 %)	0.613†
White n (%)	88 (54.0 %)	0	22 (66.7 %)	66 (50.8 %)	0.102†
Age at diagnosis of hemophilia A - years, median (IQR)	0.9 (0.5–1.4)	6	0.8 (0.6–1.4)	0.9 (0.5–1.4)	0.687‡
Severe disease n (%)	145 (91.2 %)	4	30 (90.9 %)	115 (91.3 %)	0.948†
*F8* genotype n (%)	142 (87.1 %)	21			
High-risk mutation	129 (90.9 %)		30 (100.0 %)	99 (88.4 %)	0.050†
Inversion of intron 22	69 (48.6 %)				
Inversion of intron 1	3 (2.1 %)				
Frameshift	20 (14.1 %)				
Nonsense	24 (16.9 %)				
Large deletion	13 (9.2 %)				
Low-risk mutation	13 (9.1 %)	0	0 (0.0 %)	13 (11.6 %)	
Missense	7 (4.9 %)				
Splice-site	6 (4.2 %)				
Inhibitor characteristic					
Age at inhibitor diagnosis - years median (IQR)	3.4 (1.6–9.3)	0	3.1 (1.6–7.1)	3.7 (1.6–10.1)	0.660‡
Interval between diagnoses of hemophilia A and inhibitor - years median (IQR)	2.0 (0.7–6.2)	6	1.6 (0.7–5.2)	2.1 (0.7–6.4)	0.666‡
High-response inhibitor n (%)	157 (96.3 %)	0	32 (97.0 %)	125 (96.2 %)	0.824†
Historic inhibitor peak - BU/mL median (IQR)	41.8 (15.0–97.0)	1	57.0 (20.0–202.0)	41.6 (14.0–81.4)	0.102‡
Inhibitor titer immediately before ITI - BU/mL median (IQR)	6.0 (2.8–12.8)	0	5.6 (1.5–12.8)	6.0 (2.9–12.8)	0.522‡
ITI characteristic					
Age at start - years median (IQR)	7.0 (2.5–19.7)	0	7.4 (2.2–16.2)	6.7 (2.6–20.1)	0.833‡
Interval between inhibitor diagnosis and start - years median (IQR)	1.7 (0.6–8.9)	0	2.8 (0.6–8.9)	1.5 (0.6–9.0)	0.489‡
pdFVIII as first factor n (%)	98 (60.5 %)	1	17 (51.5 %)	81 (62.8 %)	0.237†
Need for intensification n (%)	64 (39.3 %)	0	14 (42.4 %)	50 (38.5 %)	0.677†
Need for prophylaxis with bypassing agents n (%)	107 (65.6 %)	0	26 (78.8 %)	81 (62.3 %)	0.075†
Bleedings/year median (IQR)	3.4 (1.7–6.8)	2	3.5 (1.7–10.7)	3.2 (1.7–6.4)	0.455‡
Inhibitor peak - BU/mL median (IQR)	25.3 (5.6–121.6)	2	23.6 (6.4–126.0)	25.3 (4.6–121.6)	0.880‡
Success n (%)	112 (68.7 %)	0	24 (72.7 %)	88 (67.7 %)	0.577†
Duration - years median (IQR)	2.6 (1.7–3.1)	1	2.6 (1.9–3.2)	2.6 (1.6–3.1)	0.710‡

† Pearson's *χ*^2^ test.

‡ Mann-Whitney's *U* test BU: Bethesda unit; *F8*: gene encoding the clotting factor VIII; IQR: interquartile range; ITI: immune tolerance induction; pdFVIII: plasma-derived factor VIII.

Thirty-three (20.2 %) patients were submitted to 45 surgical procedures during ITI therapy (Table 1, Table 2) with insertions and removals of central lines being the most prevalent procedures (*n* = 18; 40.0 %), followed by dental procedures (*n* = 13; 29.0 %), and radiosynoviortheses (*n* = 9; 20.0 %). One patient (2.2 %) underwent each of the following procedures: external ventricular drain insertion, orchiopexy, joint corticosteroid infiltration, nasal cauterization, and cataract surgery. At the discretion of the physicians, all surgeries were performed under bypassing agent coverage. Patient, hemophilia, inhibitor, and ITI characteristics were similar between those who underwent surgical procedures and those who did not (Table 1). The success rates of ITI in these two groups were 72.7 % and 67.7 % (p-value = 0.577), respectively. Among individuals who successfully completed ITI, the time from inhibitor development until tolerance induction was similar for individuals who underwent surgeries (1.6 years; IQR: 0.9–2.0 years) and those who did not (1.6 years; IQR: 0.8–2.3 years; p-value = 0.717). The median total duration of ITI of individuals who had success (*n* = 112) and underwent surgeries was 2.3 years (IQR: 1.8–3.0 years) and for those who had success and did not undergo surgeries it was 2.1 years (IQR: 1.3–2.9 years; p-value = 0.192).

**Table 2:** Type and number of surgeries performed in people with hemophilia A and inhibitors during immune tolerance induction.

Procedure	Number (%)
Central venous access (both insertions and removals)	18 (40.0 %)
Dental procedures (tooth extractions and others)	13 (29.0 %)
Radiosynoviorthesis	9 (20.0 %)
External ventricular drain insertion	1 (2.2 %)
Orchiopexy	1 (2.2 %)
Joint corticosteroid infiltration	1 (2.2 %)
Nasal cauterization	1 (2.2 %)
Cataract surgery	1 (2.2 %)

## Discussion

The outcomes of surgical procedures association with ITI were evaluated in a large (*n* = 163) cohort of hemophilia A patients. We found that surgical procedures performed during ITI were not associated with therapy outcome.

About 20 % of participants of this study were submitted to 43 (96 %) minor surgeries and two major surgeries during ITI therapy with central venous catheter and dental procedures accounted for almost 70 %. The scarcity of major surgeries during ITI is expected due to the complexity and risk of bleeding and other complications. Indeed, until a few years ago, physicians often avoided elective surgical procedures in hemophiliacs during ITI because bleeding events were often difficult to manage and due to the inflammatory response to surgical trauma.^9^, ^10^, ^11^ However, central lines are frequently needed to facilitate infusions of both FVIII and bypassing agents.^12^ Although, procedures such as radiosynoviorthesis and cataract surgery can be postponed, the insertion of an external ventricular drain and nasal cauterization may be characterized as emergencies.

This study has several strengths. A large number of people, who underwent ITI with the same protocol, were enrolled without any selection based on the probability of success or failure of induction. In addition, participants were well characterized with few missing data. To the best of our knowledge, this is the first study to report on surgical procedures of patients while undergoing ITI.

Our study has some limitations worth mentioning. Firstly, we did not have access to perioperative data, such as the bypassing agent regimens used, severity and frequency of bleeding episodes, and length of hospital stay for those who required hospitalization. Secondly, we did not have data about inhibitor titers before and after surgery which would have helped us to determine if the procedure had elicited the immune response. Thirdly, since most of the surgical procedures evaluated in the current study are considered minor (e.g., central line insertion/removal),^8^ we could not rule out that major surgeries may have an impact on induction therapy. However, the categorization of the complexity of surgical procedures, mainly of these patients, is far from being determined.^8^^,^^13^ Fourthly, the results of this study mainly apply to three types of procedures (central venous access insertions and removals, dental procedures and radiosynoviorthesis) accounting for 89 % of all cases and so it may not be possible to generalize the conclusion to other types of minor surgeries. Procedures were classified according to a previous report that reviewed the classifications adopted for different procedures in these patients.^8^ The other proposed classification requires detailed hemostatic coverage and monitoring^14^ which were not feasible in this study.

## Conclusion

No influence of minor surgical procedures on the outcome of ITI therapy was identified in these patients. However, we reiterate that the numbers of some types of surgical procedures were small and may limit generalizability.

## Conflicts of interest

RMC received speaker fees from Bayer, NovoNordisk Hoffman-La Roche, and Takeda; consultancy fees from Hoffman-La Roche and Takeda, and scientific event grants from Bayer, NovoNordisk, Hoffman-La Roche, and Takeda. MMD and SMR declare they have no competing interests which might be perceived as posing a conflict of bias.
